# *S. mansoni* Trapping in Lungs Contributes to Resistance to Reinfection

**DOI:** 10.3389/fimmu.2015.00186

**Published:** 2015-04-21

**Authors:** Paul Mark Knopf, Parmjeet Behl Suri

**Affiliations:** ^1^Department of Molecular Microbiology and Immunology, Brown University, Providence, RI, USA; ^2^BCR Diagnostics, Chandler, AZ, USA (retired)

**Keywords:** *S. mansoni*, portacaval shunt, self-cure, resistance-to-reinfection, F2x (sera from twice-infected Fisher rats), W2x (sera from twice-infected Wistar–Furth rats), permissive vs. non-permissive hosts

## Abstract

Worm transplantation studies show that physiological and reproductive status of the worm is influenced by the microenvironment of the host and critical for vaccine design. Worm migration studies in rats with ^75^Se-methionine labeled cercariae demonstrated that resistance to reinfection (R/R) requires a host immune response resulting in worm death. In permissive hosts, inflammation due to anti eggs immunity leads to host death, whereas in non-permissive hosts this is not the case due to reduced egg burdens. Eggs-induced pathology and inflammatory debris resulting from immune attack on worms are important for vaccine design. Protective immune responses are perhaps induced when naïve hosts are vaccinated with either schistosome-derived molecules or attenuated cercariae as suggested by the induction of protective anti-parasite antibodies and monoclonals. However, these immunological strategies rarely produce 85–90% R/R as is achievable by portal-caval shunting. Alternatively, induction of anti-schistosoma immunity may induce portacaval shunting, seems highly unlikely although not yet tested. Differential screening with sera from twice-infected rats, protective (F2x) from Fisher vs. non-protective (W2x) from Wistar–Furth rats, was used to identify candidate vaccine antigens.

## Background

The authors’ combined research careers of over 60 years has focused on a preventive vaccine against schistosomiasis. In 1970 while Dr. Knopf was a postdoctoral fellow at the Salk Institute (California), Harvard physicist John Platt, then incumbent “Scientist In Residence,” presented a seminar entitled “What We Must Do”(1). Platt focused on the disparity between the funding and scientific resources invested on diseases of wealthier longer-lived populations such as: obesity, cancer, diabetes, and ischemic heart disease compared to diseases of the poor such as bacterial/viral and parasitic infections. Moreover, interests in anti-Vietnam war, woman’s lib, and racial inequality were also predominant in that era. So, emotionally moved and stimulated by Platt’s seminar, a group of young scientists and technicians formed study groups to explore their knowledge and potential of immunology to address this huge disparity. Topics chosen included birth control, food distribution, and infectious diseases such as schistosomiasis, malaria, and hookworm. Dr. Knopf, along with Alan Sher, Donato Cioli, and Italo Cesari, became further interested in schistosomiasis and in particular with the work of R. Smithers, R. Terry, and J. Clegg on *in vitro* culturing of Schistosoma worms (2). After visiting the schistosome research laboratory of A. MacInnis at UCLA, Dr. Knopf accepted a faculty position at Brown University in the Department of Molecular Biology and Immunology. Dr. Alferd Senft, an established researcher in the field of schistosomiasis with connections to the Rockefeller Foundation, was already a faculty member at Brown. Dr. Knopf started his research career in schistosomiasis in 1972.

At Brown, Dr. Knopf set-up the *Schistosoma mansoni* life cycle and learned to perform animal infections with help from Don Harn and F. von Lichtenberg (both at Harvard). Dr. Suri joined the laboratory in 1982 and her dissertation focused on differential screening and recombinant DNA technology to identify candidate vaccine protein antigens of *S. mansoni*. After completing postdoctoral training in liver cirrhosis, she rejoined Dr. Knopf’s laboratory as an assistant professor and continued research on the identification of T-cell epitopes on a *S. mansoni* candidate vaccine antigen using overlapping peptides and a novel prediction algorithm (3).

## Results

Dr. Knopf’s initial schistosome study (supported by Rockfeller) was an attempt to repeat data from mice (permissive hosts), in rats (non-permissive hosts). In rats, *S. mansoni* does not complete its life cycle (no eggs in stool, no worms in mesenteric veins)(4). Rats developed R/R, which was dependent on cercarial dose used in challenge infections (5). Both at 50 and 500 cercariae used for primary infection, there was significant R/R. At a primary and challenge dose of 500 cercariae/rat, there was significant R/R of 50%, but at the higher challenge dose of 5000 cercariae, no further increase in R/R was observed. We speculated this was due to either induction of immune tolerance or saturation of immune capacity by the secondary infection. In mice, however, a dose of 50 cercariae was lethal in <2 months; interestingly, a dose as high as 5000 cercariae/rat induced no morbidity at all. At 4 weeks post infection, worm yields by portal perfusion of once infected rats are maximal and so is their size. From weeks 4 to 8, worm attrition occurs with minimal worm pairing, low egg production, and poor embryo maturation to miracidia. About 50–60% of cercariae achieve this immature state, as confirmed by the use of radiolabeled worms (6). In mice, worms continue to grow rapidly until 6 weeks, with pairing occurring earlier and female worms laying eggs. The number of eggs is far fewer in infected rats as is worm yield and pairing by 6 weeks (~10% of mouse yields). We developed a modified assay to collect lung-stage worms, adding HEPES (7) before incubation of minced lungs, in order to neutralize acid generated by both worms and inflammatory responses in infected minced lung tissue. Increased somule yields were found and thus we confirmed data from mice studies (8) in rats. While worm yields increased, the percent R/R decreased from 80–90% to 50–60%.

Dr. Cioli spent a year in Dr. Knopf’s laboratory and they performed a unique series of worm transfer experiments. They assessed the survival, growth, and egg laying capacity of *S. mansoni* worms surgically transplanted from mice (permissive hosts) into rats (non-permissive hosts) or from rats to hamsters (permissive hosts) (4). Following transplantation into rats, adult mouse worms regress in size, remain in the liver, and produce small numbers of incompletely developed eggs. Conversely, transplantation of rat worms to hamster, worms increase in size, localize in the mesenteric veins, and produce numbers of eggs approaching normal hamster-grown worms within 3 weeks. These studies indicate that the physiological and reproductive status of the worm is strongly influenced by the microenvironment of the host. Furthermore, the suppression of worm growth and egg laying capacity in a non-permissive host is a reversible phenomenon since on transfer into a permissive environment; stunted worms can resume growth and oviposition.

**Figure 1:**
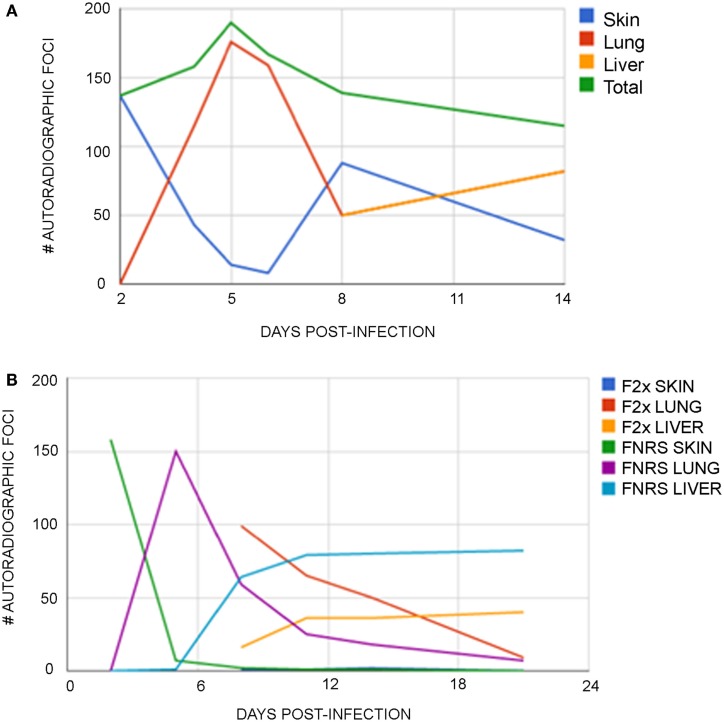
**(A)** Distribution of auto radiographic foci in normal rats at various time post infection. Rats were exposed to 200 ^75^Se- Selenomethionine-labeled cercariae on day 0. The mean number of auto radiographic foci numerated from two separate experiments are plotted. **(B)** Distribution of auto radiographic foci at various time post infection in normal and day 5 passively immunized rats. Rats were exposed to 200 ^75^Se-Selenomethionine-labeled cercariae on day 0 and a subgroup was passively immunized with F2x on day 5 post infection. The mean numbers of auto radiographic foci numerated from two separate experiments are plotted.

In collaboration with Cioli, Mangold, and Dean (6), parasite migration was studied using ^75^Se-methionine radiolabeled cercariae obtained by exposing previously infected snails to the tracer a day prior to shedding. Infected snails take up >90% of radiolabeled methionine analog in 12 h thus resulting in labeled cercariae, which were then used to infect groups of young rats (~120 g). On day 0, animals received normal Fisher rat serum (FNRS) or serum from twice-infected Fisher rats (F2x). Over the next few days, abdominal skin (infection site), as well as lung and liver tissue, was removed. Autoradiographic analysis of the pressed tissue revealed that the number of liver foci observed were nearly equal to the number of worms collected by portal perfusion (Figures 1A,B). Results confirmed that ~70% of applied cercariae attached to skin. Within 3–5 days, parasites migrated from skin to lungs with high efficiency in both normal and passively immunized rats. The total number of labeled parasites detected in skin, lungs, and liver was constant through day 5, declined to about 60% of this value by day 11 in both groups. Over the next 10 days, the rate of decline decreased significantly in normal rats, but did not change in immune rats. By day 21 post infection, nearly 50% fewer foci (labeled parasites) were detectable in livers of immune rats. The kinetics of migration from lungs to the liver differed in the two groups. In passively immunized rats with F2x, the parasites are trapped in the lungs longer as evidenced from the increased number of foci in lungs (Figure 1B). In other words, the disappearance of labeled parasites from the lungs was delayed in passively immunized rats. Labeled parasites accumulated in the liver, reaching maximal values by 11 days post infection in both groups, and remaining constant through day 21. However, half the numbers of labeled parasites were in liver of immune rats. We conclude that a subpopulation of parasites in the lungs is the target of protective immunity conferred by the F2x serum used for passive immunization. Target parasites, retained longer in lungs, were probably prevented from migrating successfully to the liver. Another parasite subpopulation migrated to the liver with normal kinetics, but was shunted out of the liver pathway and was lost forever.

Next, lung schistosomula isolated from both FNRS and F2x passively immunized rats were transferred by intravenous injection into naïve recipient rats and their continued migration from lungs to liver compared. Similar worm yields by portal perfusion from FNRS or F2x recipients were noted. We conclude that the effects of immunity molecules in F2x on somules during 1 week post infection were insignificant or reversible.

Another interpretation of the worm transfer studies could indicate a defect in worm sexual maturation in rats (6, 9). Perhaps in permissive host species, there exists a host hormone that binds to a sex-hormone receptor present in worms, but this is accomplished poorly in rats. The hormone produced by rats could partially inhibit worm maturation was studied by removal of pituitary (hypophysectomy or HYPOX) and then by removal of gonads, ovaries, thyroid, or adrenal glands. These rats were then infected with cercariae. Major finding was that worm yields and maturation improved in both *HYPOX* and in *THYROX* rats (10, 11). Thus, some step in thyroid hormone metabolism created limits on schistosome maturation and survival (Table 1).

**Table 1:** **Normal (4–8 rats) or THYROX (6 rats) per group were infected at 270 cercariae/rat and maintained daily without thyroid hormone or with T3 or T4**.

Group	Time post primary infection	Total worm yields	Male worms length (mm)	Female worms length (mm)	Sex ratio (m/f)	Weight gain (g/week)
Normal	4 weeks (5)	19.2	9.6	9.4	1.0	
	5 weeks (6–8)					41.7
	6 weeks (4)	10.5	4.5	6.0	0.75	
	7 weeks (6)	6.3	1.8	4.5	0.4	
Thyrox	7 weeks (6)	16.3	7.8	8.5	0.9	8.9
Thyrox + T3	7 weeks (6)	5.3	2.2	3.2		31.3
Thyrox + T4	7 weeks (6)	1.5	0.8	0.7	0.8	27.8

*Weighed weekly. Worms collected: number, length (mm), gender scored*.

The miracidia hatched from eggs produced in cercariae-infected THYROX rats were incubated with snails and a month later, shed cercariae collected and used to infect normal mice. About 4–6 weeks later, worms had paired and produced viable eggs. This is a Koch-like proof of the conclusion that the T3 is likely to generate, or be the source of, the inhibition of worm maturation in a non-permissive host.

During Dr. Knopf’s sabbatical leave in D. McLaren’s laboratory at Mill Hill (UK), in collaboration with G. Mitchell, worm migration was studied in inbred 129 strain of mice. Interestingly, although a permissive host, some of the inbred 129 strain of mice did not yield adult worms when infected with cercariae. Assessed at 8–10 weeks, the somules had moved from skin to lungs but failed to migrate to the portal circulation, and thus could not be collected by portal perfusion. Some worms were found in the lung capillaries involved with eosinophils. Concurrently, A. Wilson and P. Coulson described the “leaky liver” phenomenon that pre-existed in some of these 129 strain mice and was independent of infection. This was an anatomical anomaly of blood vessels that failed to yield properly located adult worms. We published our results in separately submitted papers (12, 13).

Next, our concerted efforts were focused on the use of differential screening to identify candidate vaccine antigens exclusively binding with antibodies in protective F2x but absent in non-protective W2x sera. A tegumental glycoprotein Sm25 was identified as a candidate vaccine antigen and corresponding gene (GP22) cloned. Codons 43–182 amplified by PCR and cloned in pET15b bacterial expression system. Recombinant protein r140, in combination with different adjuvants, was tested for its vaccination potential. We vaccinated mice and rats with recombinant products (3, 14–16). Despite high titer anti-r140 antibodies with protective isotypes that detected a 25 kDa surface antigen, no protection against subsequent cercarial challenge or any effect on egg yield was observed. This has led us to conclude that not all molecules produced by worms are candidate target sites. However, the differential screening technique we developed in identifying candidate vaccine antigens has proven useful in another widespread tropical disease, malaria (17).

## Conclusion

After a combined research career of 60 years, we strongly believe that we now understand something new about schistosome infection. Our research sheds light on the importance of host microenvironment in parasite attrition. Schistosomes have an enormous host range including several mammalian species such as, man and some primates, as well as rodents, and other phyla (snails for miracidia). Man and mouse appear to be good examples of *permissive* primary hosts (after infection with cercariae, viable eggs are found in feces); while *Rattus norvegicus* rats appear to be *non-permissive* hosts as cercarial infection results in absence of viable eggs in feces. Then, there are transient or *temporary permissive* hosts (permissive initially but subsequently become non-permissive) such as rhesus macaque, guinea pigs, and *Rattus rattus* (water rat, black rat). 129 strain mice are mixed. These “Transient permissive” hosts become that way by acquiring, e.g., portacaval shunts (before or during infection) or self-curing a primary infection. Also thyroidectomy can convert the rats from non-permissive to permissive state. Therefore, it seems appropriate to categorize the host in the vaccination study accordingly and be aware of the host defense mechanisms in vaccine objective.

In conclusion, one can predict two ways to end an infection by schistosoma parasites: either *via* immune attack or *via* portacaval shunting. Rats do both, *before* worms can form pairs, whereas others such as: mice, some monkey strains, and guinea pigs take longer before becoming non-permissive hosts and eliminating the parasites. Hence, the latter can be classified as *conditionally* permissive. The thyrox *R. norvegicus* rat should likely be classified as conditionally or transiently permissive.

Great leaps have been made toward schistosomiasis vaccine in the past decade, reviewed in Ref. (18–22). Calpain (Sm-p80) stands out as protective vaccine with cross species specificity, and already been tested as recombinant vaccine (hamsters and mice with Abs longevity 60 weeks in mice) and as DNA vaccine (baboons-Abs detected 5–8 years post vaccination). Coupled with purification to homogeneity, Sm-p80 is ready to be manufactured with GMP practices for clinical trials (23). Detection and testing of schistosome gut antigens related to nutrient uptake as potential vaccines is exciting (24). Use of nanoparticle gene delivery (25) and design of conformational and self-adjuvinating epitopes (26, 27) have taken the field of vaccine development to new levels. With the advances in Bioinformatics, availability of proteomic and genomic databases (28), a vaccine in the next decade could be a reality. While not identical to cases of pneumococcal bacterial or poliovirus infection, where multivalent vaccines resulted in successful vaccine, parasitic infections lead to multiple forms of the infectious agent: larval, juvenile, adult male and female worm, eggs etc., displaying unique and shared epitopes. Cocktail vaccines combining target antigens unique to different life cycle stages might give an additive protection. As pointed out by McManus and Loukas (18), putative resistant (PR) – a well defined cohort of individuals in Brazil, who display natural resistance to schistosome infection despite years of exposure to *S. mansoni*, should be studied further. They might hold a clue and give new insight to vaccine development.

## Conflict of Interest Statement

The authors declare that the research was conducted in the absence of any commercial or financial relationships that could be construed as a potential conflict of interest.
